# Identification of a differentiation-related prognostic nomogram based on single-cell RNA sequencing in clear cell renal cell carcinoma

**DOI:** 10.1038/s41598-022-15206-6

**Published:** 2022-06-29

**Authors:** Zhi-Nan Xia, Jing-Gen Wu, Wen-Hao Yao, Yu-Yang Meng, Wen-Gang Jian, Teng-Da Wang, Wei Xue, Yi-Peng Yu, Li-Cheng Cai, Xing-Yuan Wang, Peng Zhang, Zhi-Yuan Li, Hao Zhou, Zhi-Cheng Jiang, Jia-Yu Zhou, Cheng Zhang

**Affiliations:** 1 Department of Urology, The First Affiliated Hospital of Harbin Medical University, Harbin, 150001 China; 2 Department of Urology Andrology, Women’s Hospital School of Medicine Zhejiang University, Hangzhou, 310006 China; 3 Department of Urology, The Fourth Affiliated Hospital Zhejiang University School of Medicine, Yiwu City, 322000 China; 4 Xinjiang Second Medical College, Karamay City, 834000 China; 5 Zhejiang University School of Medicine, Hangzhou, 310058 China

**Keywords:** Cancer, Computational biology and bioinformatics

## Abstract

Renal cell carcinoma (RCC) is a kidney cancer that is originated from the lined proximal convoluted tubule, and its major histological subtype is clear cell RCC (ccRCC). This study aimed to retrospectively analyze single-cell RNA sequencing (scRNA-seq) data from the Gene Expression Omnibus (GEO) database, to explore the correlation among the evolution of tumor microenvironment (TME), clinical outcomes, and potential immunotherapeutic responses in combination with bulk RNA-seq data from The Cancer Genome Atlas (TCGA) database, and to construct a differentiation-related genes (DRG)-based prognostic risk signature (PRS) and a nomogram to predict the prognosis of ccRCC patients. First, scRNA-seq data of ccRCC samples were systematically analyzed, and three subsets with distinct differentiation trajectories were identified. Then, ccRCC samples from TCGA database were divided into four DRG-based molecular subtypes, and it was revealed that the molecular subtypes were significantly correlated with prognosis, clinicopathological features, TME, and the expression levels of immune checkpoint genes (ICGs). A DRG-based PRS was constructed, and it was an independent prognostic factor, which could well predict the prognosis of ccRCC patients. Finally, we constructed a prognostic nomogram based on the PRS and clinicopathological characteristics, which exhibited a high accuracy and a robust predictive performance. This study highlighted the significance of trajectory differentiation of ccRCC cells and TME evolution in predicting clinical outcomes and potential immunotherapeutic responses of ccRCC patients, and the nomogram provided an intuitive and accurate method for predicting the prognosis of such patients.

## Introduction

Clear cell renal cell carcinoma (ccRCC) is a malignant tumor typically characterized as proximal renal tubular cells, and it is the major histological subtype of renal cell carcinoma (RCC), accounting for about 70% of RCC cases^1^. The incidence rate of RCC ranks third in urologic malignancies and ranks the eighth in all malignancies. About 400,000 RCC cases are diagnosed each year globally, which are related to over 175,000 deaths. In addition, the incidence and mortality rates of RCC have increased in recent decades^2,3^. More than 20% of ccRCC patients may relapse or have distant metastasis after radical nephrectomy, and ccRCC is resistant to radiotherapy and chemotherapy. Although remarkable advances have been made in the research of targeted therapy and immunotherapy, only a limited number of clinical patients have been successfully treated^4^. At present, there are no sufficiently robust molecular biomarkers that can accurately predict the prognosis of ccRCC patients and guide clinical treatment. Hence, it is highly essential to conduct in-depth research at the molecular level to more accurately estimate or predict prognosis of ccRCC patients.

Intra-tumor heterogeneity (ITH) defines the distinct genetic alterations and phenotypes between cancer cells within the same primary tumor nodule. It has been regarded as a major driver of tumor progression and adaptation, with a notable importance for validation of biomarkers and making reliable clinical decisions^5^. Moreover, a series of studies showed the deterministic nature of clonal evolution with the progression of tumors, and the evolutionary landscape in ccRCC was dominated by ITH^6–10^. Meanwhile, with the change of tumor genome, the tumor microenvironment (TME) may consequently change, and vice versa^11^. The transformation of content and properties of TME caused by the coevolution of tumor and non-tumor cells may have different effects on the prognosis and treatment of RCC^12,13^. Therefore, it is extremely essential to concentrate on a precise comprehension of ITH and evolution of TME to formulate further effective treatment strategies and reliably estimate the prognosis of ccRCC patients.

Bulk RNA sequencing (RNA-seq) technology has provided promising insights into the specific mutations or the average expression level of mixed cell populations, while it could not reveal the gene expression status of individual cells^14^. The presentation of single-cell RNA-seq (scRNA-seq) has provided a unique opportunity for a comprehensive description of genetic complexity at the cellular level, which has contributed to our understanding of ITH and evolution of TME^15^. The current study aimed to retrospectively analyze scRNA-seq data from the Gene Expression Omnibus (GEO) database, and to explore the correlation among the evolution of TME, clinical outcomes, and potential immunotherapeutic responses in combination with bulk RNA-seq data from The Cancer Genome Atlas (TCGA) database. Besides, we constructed a prognostic risk signature (PRS) and a nomogram to predict the prognosis of ccRCC patients.

## Results

### Quality control and filtering of scRNA-seq data

**Figure 1:**
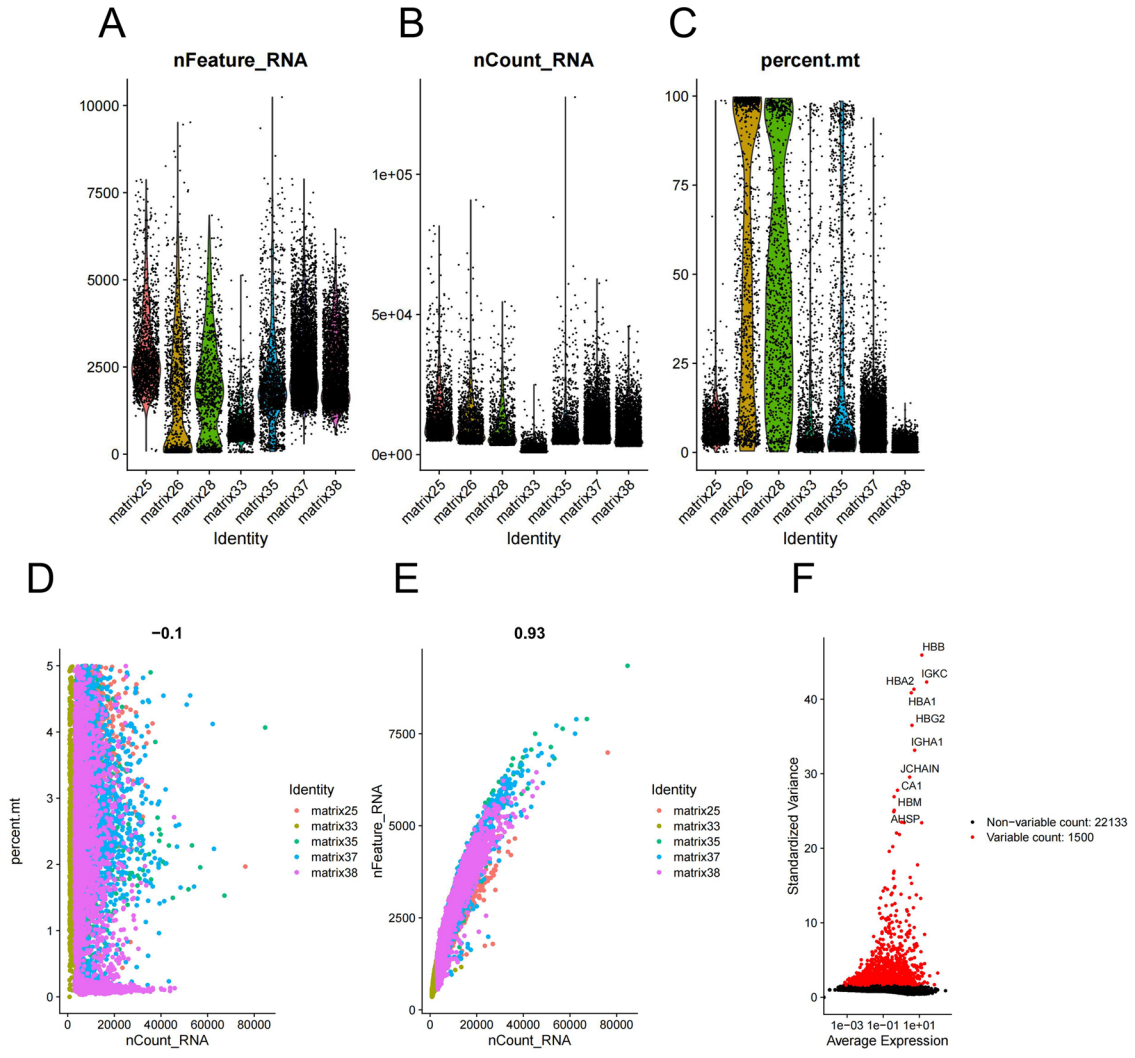
Quality control and filtering of scRNA-seq data. (**A**) The number of genes detected in 7 ccRCC samples. (**B**) Sequencing depth in each sample. (**C**) Content of mitochondria genes in each sample. High levels meant low cell activity. (**D**) A negative correlation between sequencing depth and mitochondrial gene content in 5 retrieved samples (R = −0.1). (**E**) A positive correlation between sequencing depth and total intracellular sequences in 5 retrieved samples (R = 0.93). (**F**) Volcano plot illustrated genes fluctuating in all samples. The first 1,500 genes marked in red had high variations, and the names of the top 10 genes are presented. Our scRNA-seq data is from GEO database (https://www.ncbi.nlm.nih.gov/geo/query/acc.cgi?acc=GSE159115/).

In our study, 17,665 scRNA-seq data from 7 ccRCC samples were acquired from GSE159115 (Fig. 1A,B). Mitochondrial gene expression is a classical quality control index of single-cell analysis. The high expression level of mitochondrial gene may indicate poor sample quality, which means a large number of cell apoptosis or lysis. We performed quality control and abandoned two sample data (matrix 26, 28), because their mitochondrial gene contents were extremely large (Fig. 1C). After our filtering, 15,332 scRNA-seq data from 5 ccRCC samples were retrieved for further research. Sequencing depth was negatively correlated with mitochondrial content (Fig. 1D), and it was positively correlated with total intracellular sequences (Fig. 1E). A total of 22,133 genes were fluctuated in all samples, and the first 1,500 genes with the most obvious fluctuations were screened for the additional research (Fig. 1F).

### Identification of ccRCC subsets by single-cell trajectory analysis

**Figure 2:**
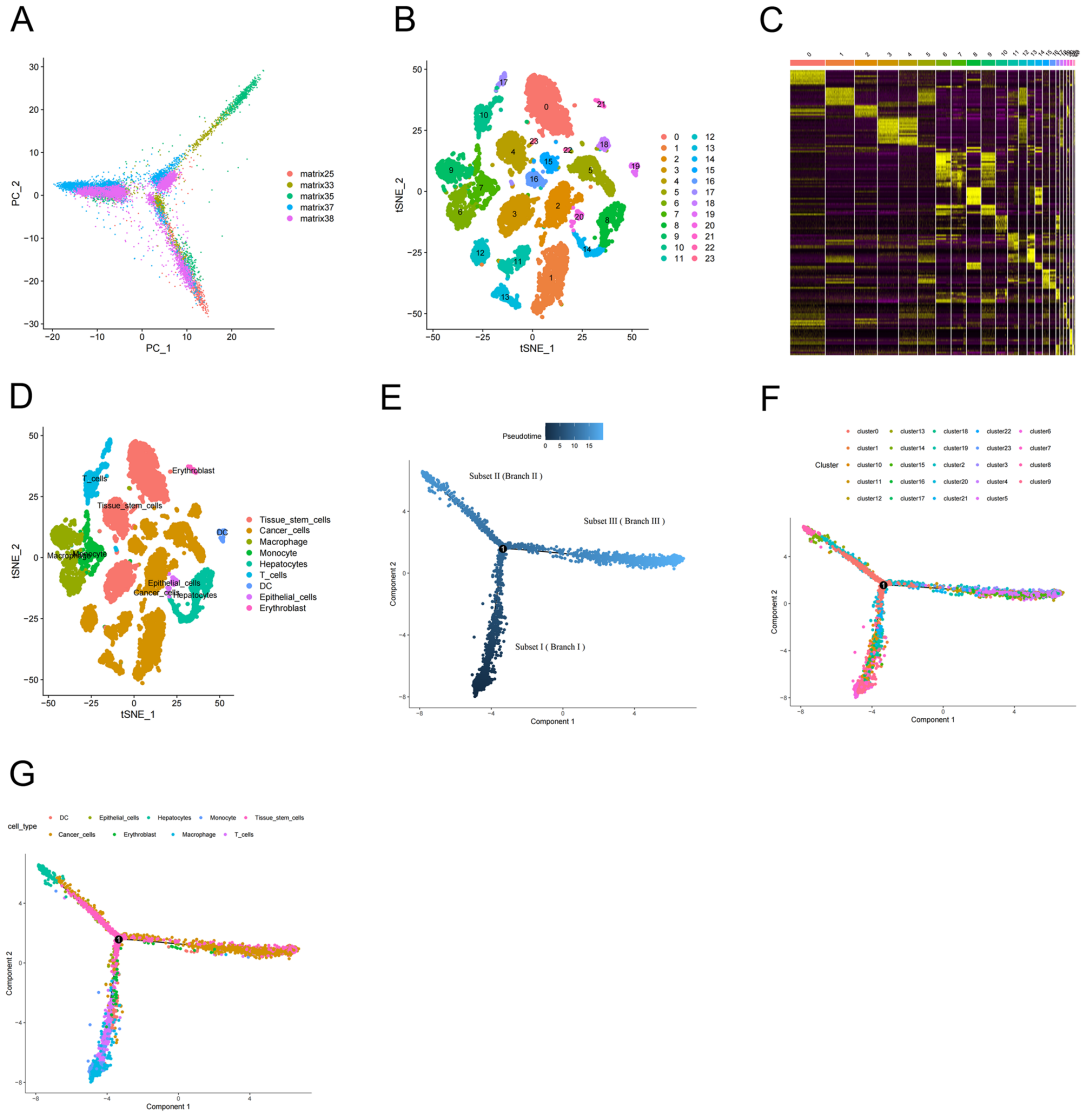
Identification of ccRCC subsets by the single-cell trajectory analysis. (**A**) PCA of scRNA-seq data for preliminary dimensionality reduction. (**B**) tSNE was used for clustering of ccRCC cells. (**C**) Heatmap of marker genes in each cluster. Yellow represents upregulated genes and purple indicates downregulated genes. (**D**) Cell-type annotation of clusters. 24 clusters were divided into 9 cell types according to marker genes. (**E**) Single-cell pseudotime analysis of three subsets. Dark blue denotes an earlier time. (**F, G**) Trajectory analysis of clusters and cell types. The dots of different colors represented the corresponding clusters or cell types, which were arranged on the pseudotime branches.

To reduce the dimensionality of scRNA-seq data preliminarily, principal component analysis (PCA) was applied (Fig. 2A). The top 15 principal components (PCs) with significant differences (P < 0.0001) were selected for further analysis. Next, we aggregated 15,332 ccRCC data into 24 clusters according to the t-distributed stochastic neighbor embedding (tSNE) algorithm (Fig. 2B), and a total of 1,292 marker genes in each cluster were identified on the heatmap (Fig. 2C). Accordingly, 24 clusters were annotated as 9 types of cells, and the results showed that abundant TME existed in ccRCC samples (Fig. 2D). The results of trajectory and pseudotime analysis demonstrated that there were three branches in differentiated cells that changed over pseudotime (Fig. 2E). The core of pseudotime analysis was an unsupervised machine learning algorithm, which could infer the time and sequence of cell differentiation according to the single-cell expression data^16^. In addition, the results showed the relationship among cell trajectories, cell types, and clusters, which could infer the changes of TME by the corresponding cell type and time sequence (Fig. 2F,G). It was concluded that the most primitive components in TME are various immune cells, in which the number of stem-like cells subsequently increases. The stem-like cells seemed to be the precursors of tumor cells^16^. With the extension of time trajectory, the proportion of tumor cells showed a gradual increasing trend, which was in line with the law of tumor development.

### GO and KEGG enrichment analyses of differentiation-related genes (DRGs) in three subsets

**Figure 3:**
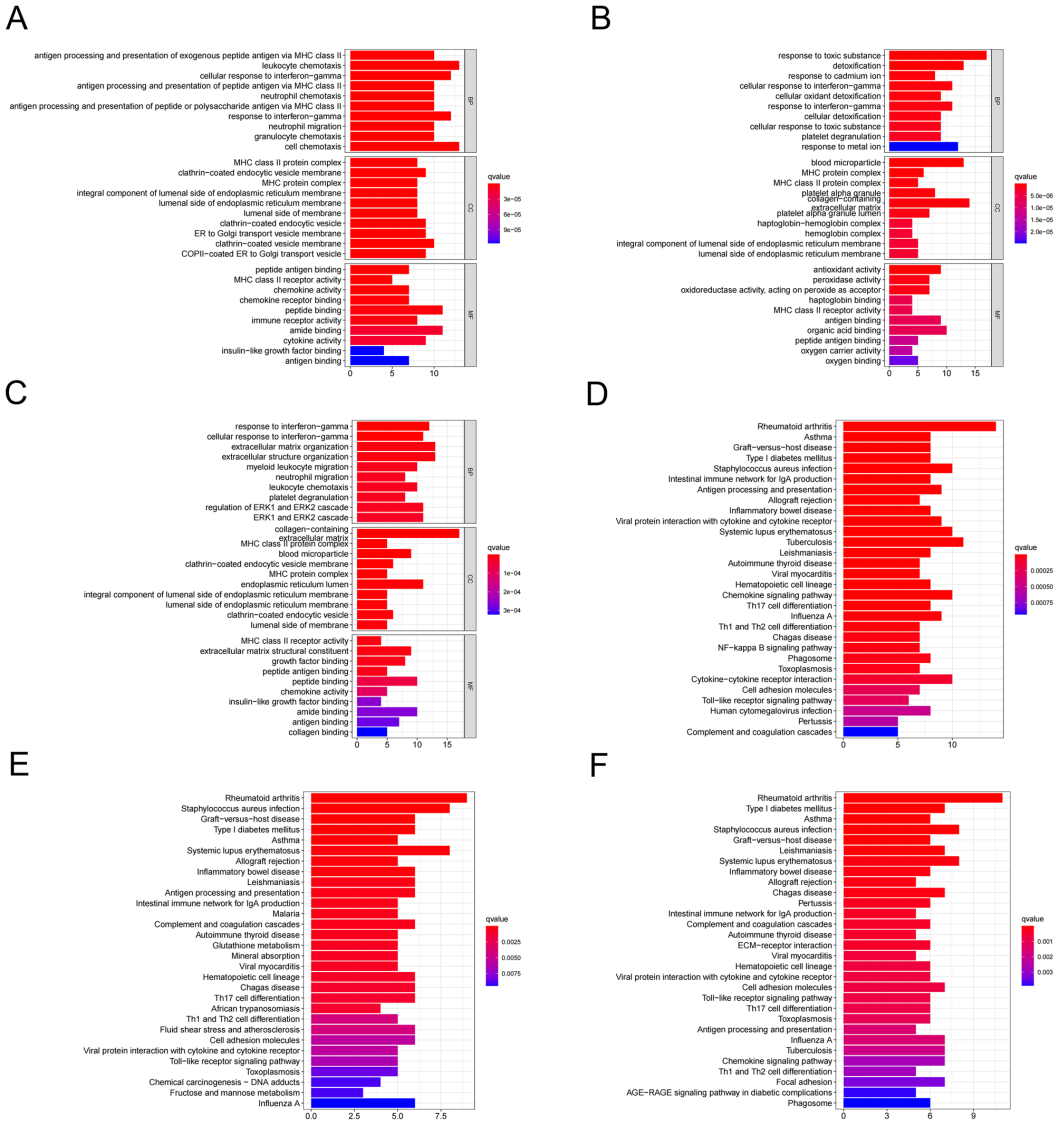
GO and KEGG enrichment analyses of DRGs in three subsets. (**A–C**) GO functional analysis of DRGs in subsets I-III. (**D–F**) KEGG pathway enrichment analysis of DRGs in subsets I-III.

We identified differentially expressed genes of each trajectory with a distinct differentiation, which were considered as DRGs. To determine the functions of DRGs in the three subsets, the Kyoto Encyclopedia of Genes and Genomes (KEGG) pathway enrichment and Gene Ontology (GO) analyses were conducted. GO functional analysis indicated enrichment of DRGs in the three subsets mainly included response to interferon − gamma, MHC protein complex, and antigen binding pathway. Respectively, DRGs in subset I were mainly enriched in antigen processing and presentation and chemotaxis of various immune cells, those in subset II were involved in response to toxic substances and detoxification, and those in subset III were related to extracellular organization, migration of immune cells, and regulation of ERK1 and ERK2 cascades (Fig. 3A–C). Furthermore, the pathways involved in antigen processing and presentation, chemokine signaling pathway, differentiation of helper T cells, and some immune-related and autoimmune diseases were identified in the three subsets by the KEGG pathway enrichment analysis (Fig. 3D–F).

### Identification of DRG-based molecular subtypes in ccRCC samples from TCGA-KIRC database

**Figure 4:**
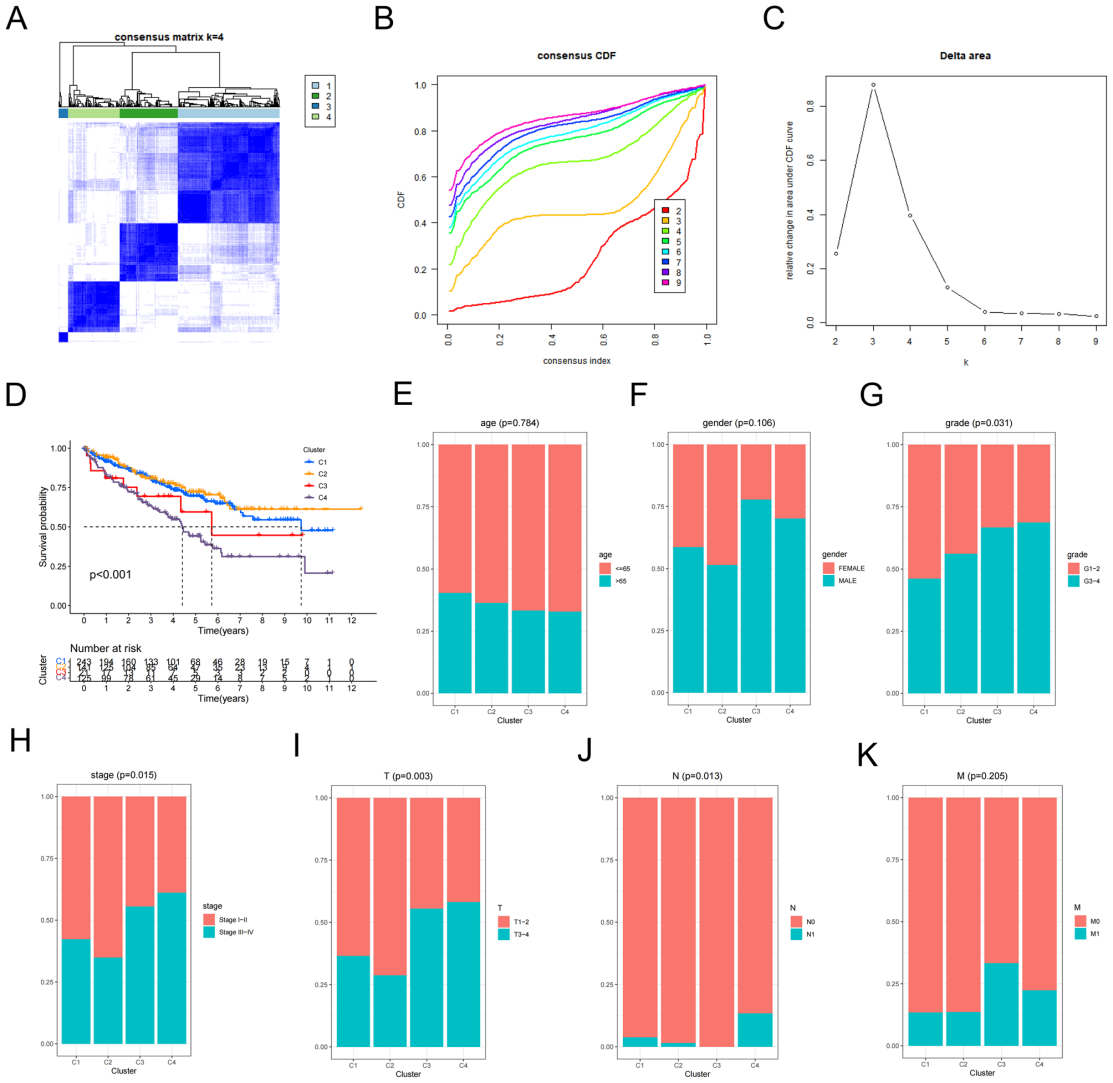
Identification of DRG-based molecular subtypes and clinical analyses. (**A–C**) Four molecular subtypes were identified at a clustering threshold of K_max_ = 9. (**D**) The Kaplan–Meier survival plot for four molecular subtypes (P < 0.001). (**E–K**) Proportions of clinicopathological features (age, gender, grade, stage and TNM) among four molecular subtypes. P < 0.05 was considered statistically significant. The bulk RNA-seq data and clinicopathological data of ccRCC samples were accessed from TCGA data base (https://portal.gdc.cancer.gov/).

The clinical data and transcriptome of 539 ccRCC samples were harvested from TCGA-KIRC database, and DRG-based consensus cluster analysis was conducted. Four molecular subtypes in ccRCC samples were identified at a clustering threshold of K_max_ equal to 9, based on the DRG intersection of three branches (Fig. 4A–C). The Kaplan–Meier survival plot confirmed the significant correlation between molecular subtypes and survival data, and the curve illustrated that subtype II (C2) had the longest overall survival (OS), followed by subtype I (C1), subtype III (C3), and subtype IV (C4) (Fig. 4D). To determine whether molecular subtypes are dependent on clinicopathological features, we performed clinical correlation analyses using the Chi-square test. As shown in Fig. 4G–J, molecular subtypes were related to the Fuhrman’s grades, clinical stage, tumor size (TS), and lymph node metastasis (N). The number of patients with high Fuhrman’s grades increased from C1 to C4, and similar to the survival curve, clinical stage and TS had the highest association with C2, followed by C1, C3, and C4. Nevertheless, molecular subtypes were not significantly associated with age, gender, and distant metastasis (M) (Fig. 4E, F, K).

### Analysis of TME and TIICs in four molecular subtypes

**Figure 5:**
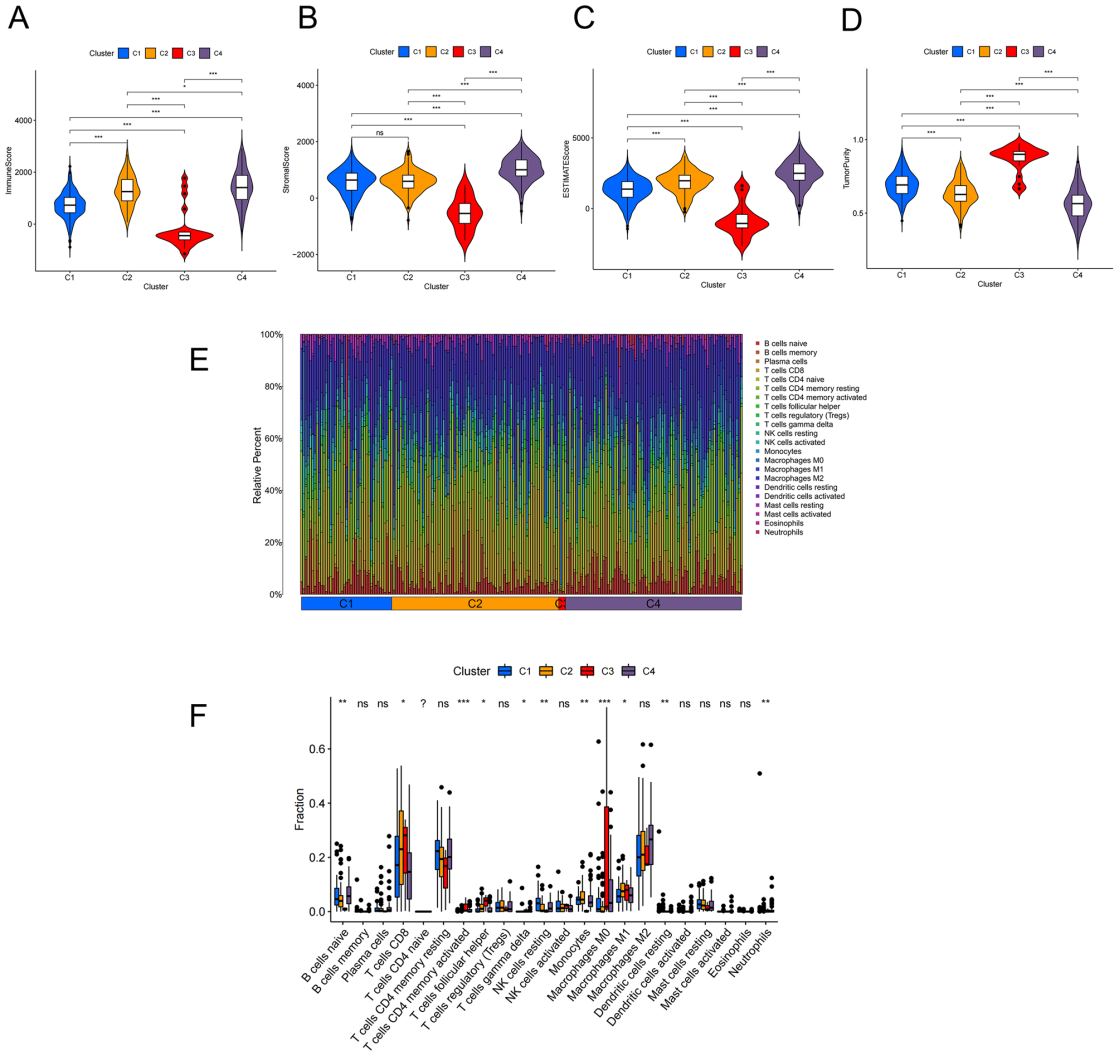
Comprehensive analysis of TME and TIICs in four molecular subtypes. (**A–D**) ImmuneScore, StromalScore, ESTIMATEScore, and tumor purity in four molecular subtypes. (**E**) The contents of 22 TIICs in each sample are displayed according to four molecular subtypes. (**F**) Differential expression analysis of TIICs in four molecular subtypes. (*P < 0.05, **P < 0.01, ***P < 0.001, ns: not significant).

To evaluate immune/stromal components and tumor purity for each molecular subtype, the ESTIMATE algorithm was employed. A higher ImmuneScore/StromalScore indicated a greater number of immune/stromal components in TME, and the sum of these two scores was indicated by the ESTIMATEScore (representing a comprehensive scale for the couple of components in TME). The higher the ESTIMATEScore, the higher the non-tumor component, and the lower the corresponding tumor purity. Except for C3 that had the lowest number of immune/stromal components, the number of immune/stromal components increased from C1 to C4 (Fig. 5A–C). Correspondingly, contrary to the above-mentioned results, the tumor purity decreased from C1 to C4, except for C3 that had the highest level of tumor purity (Fig. 5D). Using the CIBERSORT algorithm, we performed further analyses for verification of the correlation of molecular subtypes with the TME. The contents of 22 tumor-infiltrating immune cells (TIICs) in each sample were displayed according to four molecular subtypes (Fig. 5E). Differential expression analysis of TIICs demonstrated that the differences in 11 immune cells were statistically significant among four molecular subtypes. The M0 macrophages and CD4 memory T cells were significantly activated, and the higher infiltration degree of these cells was correlated with a worse OS. Among the statistically significant immune cells, CD8 + T cells were the most abundant, and a higher infiltration was associated with a longer OS, except for C3 subtype (Fig. 5F). In the study of TME, the results of C3 subtype significantly differed from other subtypes. This may be related to the fact that the sample size of C3 subtype was relatively smaller, reducing the power of statistical analysis.

### Comprehensive analysis of ICGs in four molecular subtypes

**Figure 6:**
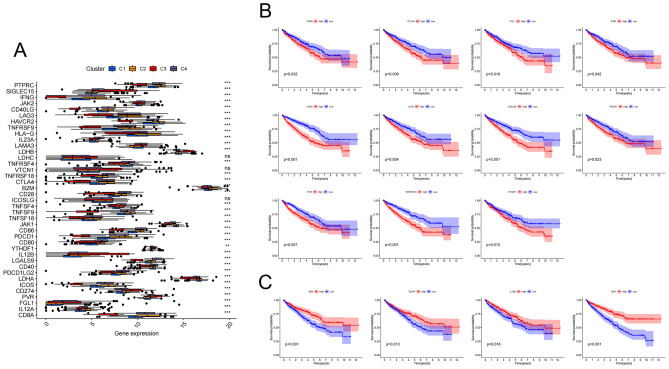
Comprehensive analysis of ICGs in four molecular subtypes. (**A**) Differential expression analysis of 39 ICGs. (**B**) Kaplan–Meier survival plots of ICGs with a poor prognosis. (**C**) Kaplan–Meier survival plots of ICGs with a promising prognosis. The blue curve represents the low-expression group, and the red curve represents the high-expression group.

We obtained 39 confirmed immune checkpoint genes (ICGs) from previous studies and analyzed their differences among the four molecular subtypes^17–26^. There was a significant difference in almost all ICGs (Fig. 6A) among those subtypes. Survival analysis was performed to find out the correlation between the expression levels of these ICGs and the prognosis. According to the median expression level of these ICGs, the samples were divided into high-expression group and low-expression group, and Kaplan–Meier plots showed that the expression levels of CD80, CTLA4, FGL1, IFNG, IL23A, LAG3, LGALS9, PDCD1, PVR, TNFRSF18, and YTHDF1 were associated with a short OS (Fig. 6B), while B2M, CD274, IL12B, and JAK1 predicted a longer OS (Fig. 6C). These results not only provided a reasonable molecular basis for different levels of prognosis in the four subtypes, but also could potentially guide immunotherapy.

### Identification, evaluation, and validation of a prognostic risk signature (PRS)

**Figure 7:**
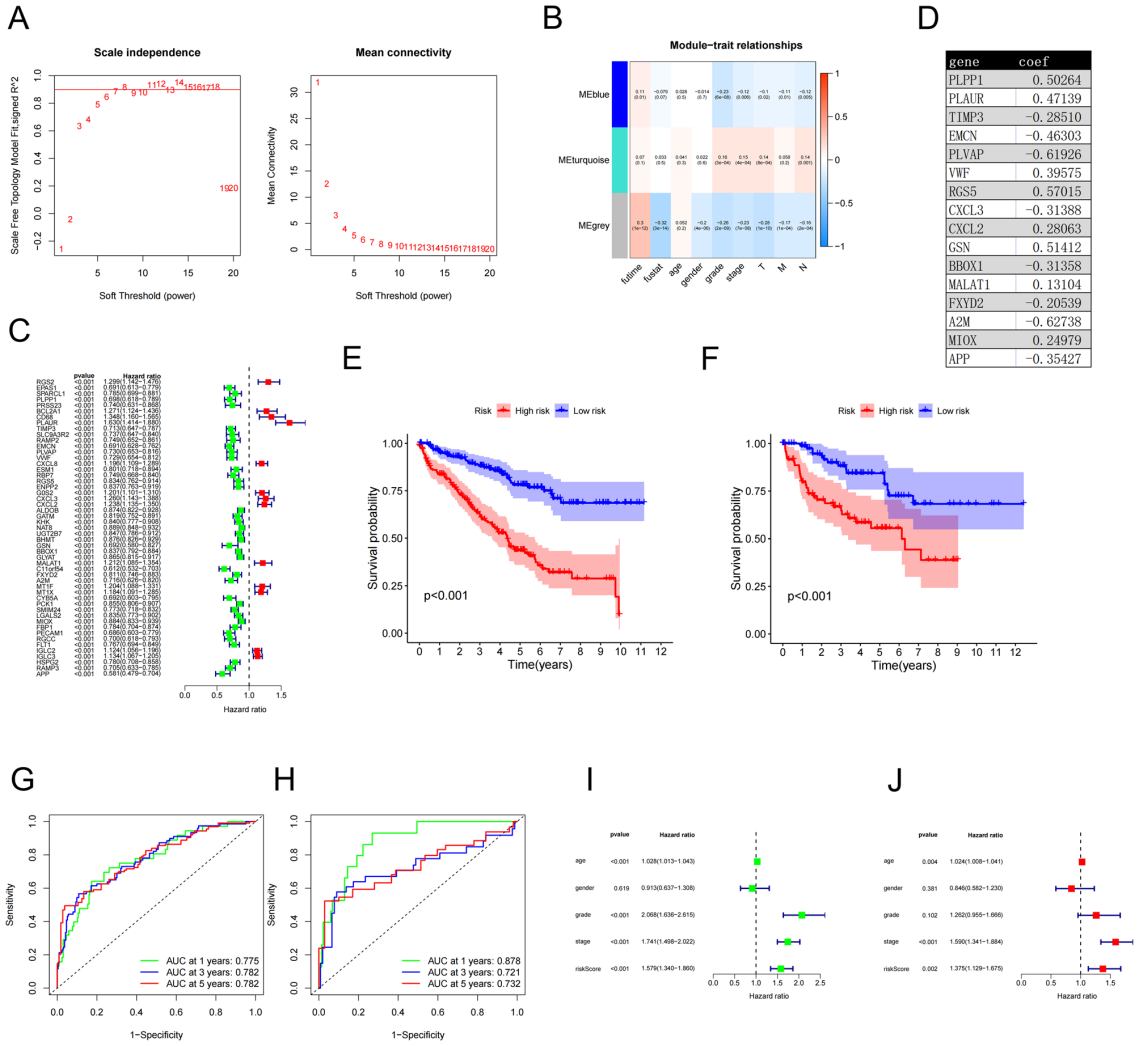
Identification, evaluation, and validation of a prognostic risk signature. (**A,B**) Three modules were accessed with power = 7 based on WGCNA, and the blue module was chosen for further analysis (P (MEblue-futime) < 0.05, P (MEblue-grade) < 0.05). (**C**) A forest plot illustrating the results of univariate analysis of DRGs. (**D**) The genes used to establish the model and their corresponding coefficients. (**E, F**) Kaplan–Meier survival plot displaying the results of survival analysis in the high-risk and the low-risk groups in the train and the test cohorts (the left-sided plot belongs to the train cohort and the right-sided one belongs to the test cohort). (**G, H**) The values of area under the ROC curves for predicting 1-, 3-, and 5-year survival in the train and test cohorts. (the left-sided plot belongs to the train cohort and the right-sided one belongs to the test cohort). (**I**) Univariate analysis of RS and clinicopathological characteristics. (**J**) Multivariate logistic regression analysis of RS and clinicopathological characteristics.

We enrolled 149 DRGs, and three modules were assessed by weighted gene co-expression network analysis (WGCNA), of which one module (MEblue) was related to both survival and the Fuhrman’s grade of ccRCC samples (Fig. 7A,B). We abandoned the grey module for further research, because the genes of gray module were discrete and not co-expressed, representing genes that did not belong to any module. Then, 51 prognostic DRGs were further screened by univariate and multivariate logistic regression analyses (Fig. 7C). We divided TCGA ccRCC samples into the train cohort and the test cohort with a ratio of 7:3, and the training cohort was used to build the signature and the test cohort was used to test the effect of it. Finally, a PRS, consisting of 16 DRGs, was identified by multivariate Cox analysis, and the risk score (RS) of each sample was calculated according to the relative coefficient and gene expression level (Fig. 7D).

RS = (0.50264 * expression level of PLPP1) + (0.47139 * expression level of PLAUR) + (−0.28510 * expression level of TIMP3) + (−0.46303 * expression level of EMCN) + (−0.61926 * expression level of PLVAP) + (0.39575 * expression level of VWF) + (0.57015 * expression level of RGS5) + (−0.31388 * expression level of CXCL3) + (0.28063 * expression level of CXCL2) + (0.51412 * expression level of GSN) + (−0.31358 * expression level of BBOX1) + (0.13104 * expression level of MALAT1) + (−0.20539 * expression level of FXYD2) + (−0.62738 * expression level of A2M) + (0.24979 * expression level of MIOX) + (−0.35427 * expression level of APP).

We divided the two cohorts into low-risk group and high-risk group according to the median RS. The OS in the low-risk group was significantly longer than that in the high-risk group in the two cohorts (Fig. 7E, F). In addition, the values of area under the receiver operating characteristic (ROC) curve for predicting 1-, 3-, and 5-year survival in the train cohort were 0.775, 0.782, and 0.782, respectively, and 0.878, 0.721, and 0.732 in the test cohort, respectively (Fig. 7G,H). In the train cohort, the results of univariate analysis revealed that Fuhrman’s grade, TNM stage, and RS could independently affect the prognosis (Fig. 7I). The results of multivariate logistic regression analysis demonstrated that TNM stage, and RS were independent prognostic factors for ccRCC patients (Fig. 7J). Since the Hazard ratio of age was approximately equal to 1, although the p value was statistically significant, we did not include it into independent prognostic factors. The increased RS and these clinicopathological characteristics were associated with a poor prognosis.

### Correlation between RS and immune characteristics

**Figure 8:**
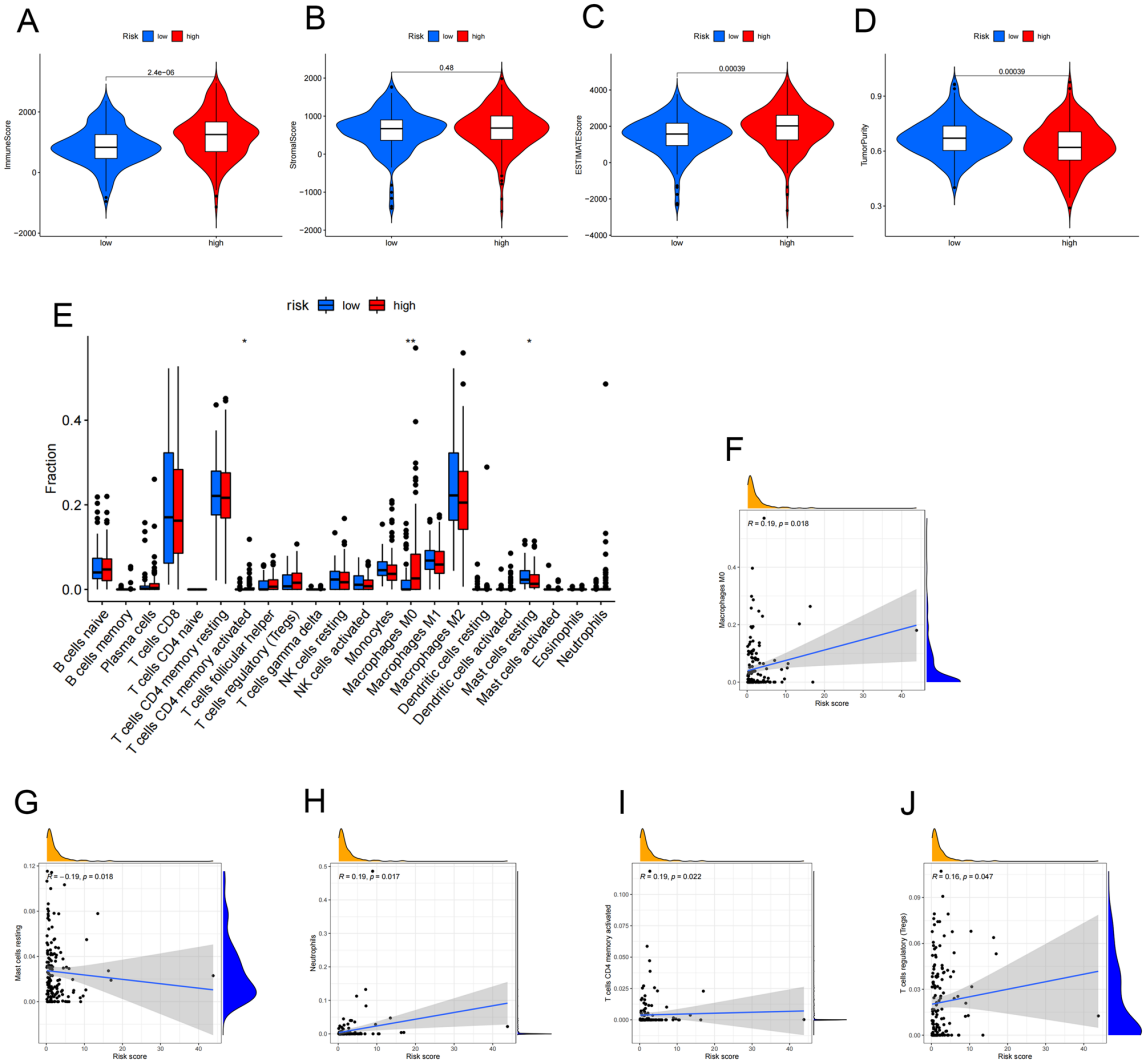
Correlation between RS and immune characteristics. (**A**) The relevance between high/low risk groups and the immune scores, (**B**) the stromal scores, (**C**) the ESTIMATE scores and (**D**) tumor purity. (**E**) The correlation between the RS and significant ICGs. Red circles represent positive correlation and blue circles represent negative correlation. The strength of the correlation is determined by the size of the circle. (**F–G**) The scatterplots showed the correlation among five types of TIIC and the RS with P < 0.05 by Spearman’s rank correlation test. R > 0 indicated that the two were positively correlated, and vice versa. (*P < 0.05, **P < 0.01, ***P < 0.001).

To explore the relationship between TME and RS, we divided samples of the training cohort into two groups according to the median of RS. As with the method described earlier, we calculated the immune scores, the stromal scores, the ESTIMATE scores and tumor purity, and found that the group with high RS had higher ImmuneScores, while StromalScores were not related to the grouping according to RS (Fig. 8A,B). Correspondingly, the ESTIMATEScores were higher in the high-risk group and the tumor purity was higher in the low-risk group (Fig. 8C,D). Therefore, we inferred that ccRCC patients with high RS had a high level of immune infiltration in TME. Then we implemented the correlation analysis to verify the association between 11 classic ICGs and RS. Each intersection represented the association between the two ICGs or RS, which was expressed by the color and size of the circle (Fig. 8E). There was a positive correlation between high-risk patients and CD80, CTLA4 and LAG3, while HLA-G was negatively correlated with high-risk patients. Spearman’ s rank correlation test was used to show the relationship between 22 TIICs mentioned in Fig. 5E and RS, and we found that high proportions of M0 macrophages, neutrophils, activated CD4 memory T cells and Tregs would increase the risk of an unfavorable prognosis, while high proportion of resting mast cells would reduce it (Fig. 8F–J).

### Establishment and evaluation of a nomogram for predicting survival of ccRCC patients

**Figure 9:**
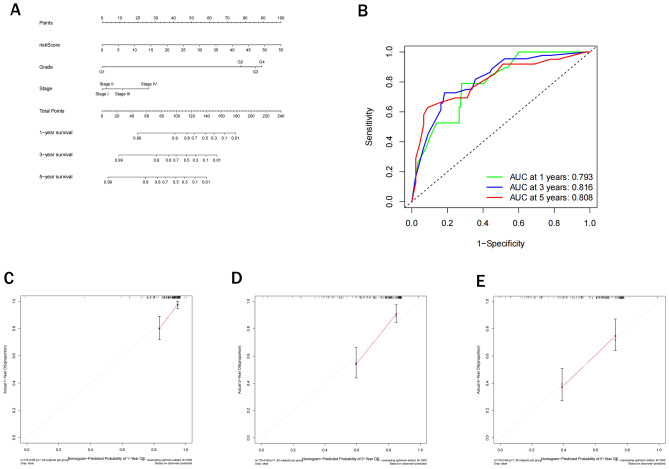
Establishment and evaluation of a prognostic nomogram. (**A**) A nomogram for predicting prognosis of patients with ccRCC. (**B**) The values of area under the ROC curves for predicting 1-, 3-, and 5-year survival. (**C–E**) 1-, 3-, and 5-year survival calibration curves of the nomogram.

Not only “age” and “gender” were not independent risk factors for ccRCC according to the results of multivariate logistic regression analysis in both training and test cohort, but also they shared small contributions to the total points in the established nomogram model. Therefore, we did not include age and gender in the construction of ultimate nomogram. Three prognostic factors, including RS and two clinicopathological characteristics (Fuhrman’s grade, and TNM stage) were combined to establish a nomogram for predicting 1-, 3-, and 5-year survival using the data of the training cohort (Fig. 9A). The values of area under the ROC curves for predicting 1-, 3-, and 5-year survival were 0.793, 0.816, and 0.808, respectively (Fig. 9B). The calibration curves were in a good agreement with the recorded values (Fig. 9C–E).

## Discussion

RCC is the third most common malignancy of urinary system and has a substantial mortality rate. Although a substantial progress has been made in the therapy of RCC, clinical outcomes of RCC patients are still unsatisfactory. Biomarkers that could accurately predict the prognosis and guide the therapy of RCC have not been fully identified and applied clinically.

To date, scholars have mainly employed RNA-seq to detect all RNA transcripts in samples for gene expression analysis. However, the objects of sequencing are often tissue samples with mixed cells or single cell lines. Therefore, scRNA-seq was proposed, and it has been extensively applied to reveal cell heterogeneity in various cell populations^27,28^. scRNA-seq data of ccRCC patients were obtained from GEO database (GSE159115) and combined with the RNA-seq data from TCGA database to accomplish our research.

Multi-region sequencing of ccRCC samples confirmed a substantial ITH, which indicated that sub-clones harboring distinct driver mutations and somatic copy number aberrations were present within primary tumors. Phylogenetic reconstruction revealed branched evolutionary tumor growth, demonstrating that sub-clones evolved with tumor progression^29–31^. In solid tumors, various immune and stromal cells in TME coevolve with the tumor progression, and the evolution of TME may influence the prognosis and treatment of tumors^11–13^. Consequently, trajectory-based differential expression analysis can assist scholars to better understand the evolution of ITH and TME. In the present study, we identified trajectories with distinct differentiation states according to the scRNA-seq data. Pseudotime analysis is a trajectory inference method from scRNA-seq data, which sorts cells along the trajectory according to the similarity of cell expression patterns, and determines the lineage structure by identifying branching events^16^. Using marker genes, 9 types of cells, including tumor and non-tumor cells, were identified using trajectory-based differential expression analysis, which enabled us to intuitively realize the testimony of TME evolution in ccRCC by combining pseudotime analysis and the location of different cell types over time.

The conventional subtypes of RCC can be divided into ccRCC, papillaryRCC (pRCC), chromophobe RCC (chRCC) and other different histological subtypes according to the location and origin of cells^32^. Molecular subtyping is to subdivide each histological subtype according to its transcriptional and gene alteration profiles. Identification of molecular cancer subtypes can be used to optimize diagnostic and treatment strategies, and promote the development of precision medicine. For instance, Motzer et al.^33^ performed an integrative multi-omics analysis on 823 RCC samples in a randomized clinical trial, and 7 molecular subsets with distinct angiogenesis, immune, cell-cycle, metabolism, and stromal programs were identified by unsupervised analysis of transcriptomic data. The molecular subsets were associated with differential clinical outcomes to angiogenesis blockade alone or with a checkpoint inhibitor. Other molecular subtypes of RCC have also been reported^34,35^, however they all have certain limitations. In the present study, we divided ccRCC samples from TCGA database into four DRG-based molecular subtypes, and OS, clinicopathological features, and the expression levels of ICGs were significantly correlated with different molecular subtypes. ccRCC is a historically immunogenic cancer, and it has been found that TIICs in TME act as a potential indicator of prognosis^36,37^, and compositions of TIICs may influence immunotherapeutic interventions^38^. Moreover, we identified 39 ICGs that could be targeted with different immunotherapeutic methods according to molecular subtypes, such as PD-L1 inhibitors and CTLA-4 inhibitors.

Because the OS of patients with different molecular subtypes can be well distinguished, we used DRGs to construct a 16-gene PRS to predict prognosis of ccRCC patients. To the best of our knowledge, this is the first DRG-based signature constructed by multivariate Cox regression analysis. Furthermore, a nomogram combining DRG-based RS and prognostic clinicopathological variables was constructed to provide a visual method for predicting prognosis of ccRCC patients. Using clinical information alone could not predict the prognosis well, but adding it to RS signature made our model more accurate and effective. Despite a high accuracy and a robust predictive performance of the nomogram, it was constructed and validated based on retrospective data from TCGA and GEO databases, and there were additional prognosis-related clinicopathological variables that could not be accessed from public databases. And according to GSE159115, samples here were all partial nephrectomy samples, which meant these are early tumors. Due to the scarcity of the data of scRNA-seq in GEO database, we couldn't get the single-cell sequencing results of advanced and metastatic RCC samples. This was also the limitation of our research. Therefore, further large-scale prospective clinical studies are required to supplement and refine the nomogram and evaluate its effectiveness and practicability.

## Conclusions

This study identified three ccRCC cell trajectories with different differentiation states based on scRNA-seq data, and it was confirmed that DRG-based molecular typing can accurately predict OS, clinicopathological features, TME, immune infiltration status, and expression levels of ICGs. The nomogram combined with RS based on DRGs and clinicopathological variables provided an intuitive and accurate method for predicting the prognosis of ccRCC patients. In conclusion, this study emphasized the significance of trajectory differentiation of ccRCC cells and TME evolution in predicting clinical outcomes and potential immunotherapeutic responses of ccRCC patients.

## Methods

### Data acquisition and processing

A total of 17,665 scRNA-seq data from 7 ccRCC samples were obtained from the GSE159115 dataset in the GEO database (https://www.ncbi.nlm.nih.gov/geo/). Then, the “PercentageFeatureSet” function was used to calculate the percentage of mitochondrial genes, and two samples were excluded because of an elevated number of mitochondrial genes, which indicated an insufficient cell viability. After data filtering, scRNA-seq data were normalized by the LogNormalize method, and the top 1,500 genes with highly variable features were identified for further analysis.

Clinical data and bulk RNA-seq data of 539 patients with ccRCC were obtained from TCGA database (https://portal.gdc.cancer.gov/), and samples with unclear clinicopathological characteristics were removed.

The R 4.0.3 programming language (https://www.r-project.org/) was employed to perform all the analyses in our study.

The types of patients and number in each database had been presented with the standard clinicopathological distribution (Supplementary Tables 1 and 2).

### Dimensionality reduction and cell annotation

Dimensions with significant separation were screened out through PCA, and the dimensions of the top 15 PCs were reduced by the tSNE algorithm to obtain principal clusters. Marker genes in each cluster were acquired and illustrated in the heatmap under the condition of log_2_ [fold-change (FC)] > 2 and false discovery rate (FDR) < 0.05 by the “pheatmap” package. Clusters were annotated into 9 types of cells based on marker genes by the “SingleR” package.

### Single-cell trajectory analysis

The single-cell trajectory analysis was undertaken by the “Monocle” package, and differentially expressed genes of each trajectory with a distinct differentiation were designated as DRGs.

### GO and KEGG enrichment analyses

GO and KEGG pathway enrichment analyses of DRGs in the three subsets were conducted by the “clusterProfiler”, “enrichplot”, “org.Hs.eg.db”, and “ggplot2” packages. P < 0.05 was considered statistically significant.

### DRG-based molecular subtypes

The ‘ConsensusClusterPlus’ package was used for consensus clustering to quantify the number of unsupervised subtypes. The K-means algorithm and cumulative distribution function (CDF) were utilized to determine the optimal number of subtypes, and 50 iterations with K_max_ equal to 9 were considered for stable subtypes.

### Survival and differential expression analyses of subtypes using clinicopathological features

The Kaplan–Meier plotter was used to plot the survival curve, and the proportion of clinicopathological features in each molecular subtype was drawn by the “ggplot2” package. P < 0.05 was considered statistically significant.

### Analysis of TME, TIICs, and ICGs

The ESTIMATE algorithm was employed to calculate the ratio of immune/stromal component and tumor purity in TME for each molecular subtype. The CIBERSORT algorithm was used to estimate contents of 22 TIICs in each sample. Besides, 39 validated ICGs were summarized and examined by differential expression analysis, and then, the survival analysis was carried out to investigate prognosis.

### Construction of WGCNA and correlation analysis

The WGCNA was constructed using the gene expression data. Clinically meaningful modules were identified using Pearson correlation analysis to identify correlations between modules and clinical features, and key modules related to differentiation of ccRCC cells were selected for subsequent analysis.

### Generation and validation of the PRS

Herein, 70% of TCGA ccRCC samples were taken as the training set, and the 30% were considered as the validation set for generation and validation of the PRS. The DRGs in key modules of Weighted Gene Correlation Network Analysis (WCGNA) were analyzed by univariate analysis (P < 0.001), and the remaining DRGs were analyzed by multivariate logistic regression analysis to generate a DRG-based PRS. The RS was calculated as follows:

$${\text{Risk}}\;{\text{score}} = \sum {\text{ Cox}}\;{\text{coefficient}}\;{\text{of}}\;{\text{gene}}\;G_{i} \times {\text{expression}}\;{\text{value}}\;{\text{of}}\;{\text{gene}}\;G_{i} .$$

Furthermore, we conducted the Kaplan–Meier analysis, ROC curve analysis, and univariate and multivariate logistic regression analyses to verify the accuracy of the classifier.

### Construction and validation of the prognostic nomogram

Prognostic variables were combined into the nomogram to predict 1-, 3-, and 5-year survival. ROC and calibration curves were plotted to evaluate the predictive performance and accuracy of the nomogram.

## Supplementary Information

Supplementary Information 1.

Supplementary Information 2.

## Data Availability

The scRNA-seq data of ccRCC samples were accessed from GEO database (GSE159115, https://www.ncbi.nlm.nih.gov/geo/). The bulk RNA-seq data of ccRCC samples were accessed from TCGA data base (https://portal.gdc.cancer.gov/). Data will be available from the corresponding author upon reasonable request. All the procedures were performed in accordance with the relevant guidelines and regulations.

## Abbreviations

ccRCC: Clear cell renal cell carcinoma
DRG: Differentiation related gene
GEO: Gene Expression Omnibus
GO: Gene Ontology
ICG: Immune checkpoint gene
ITH: Intra-tumor heterogeneity
KEGG: Kyoto Encyclopedia of Genes and Genomes
OS: Overall survival
PCA: Principal component analysis
PRS: Prognostic risk signature
RCC: Renal cell carcinoma
ROC: Receiver operating characteristic
RS: Risk score
scRNA-seq: Single-cell RNA sequencing
TCGA: The Cancer Genome Atlas
TIIC: Tumor-infiltrating immune cell
TME: Tumor microenvironment
tSNE: T-distributed stochastic neighbor embedding
WGCNA: Weighted gene co-expression network analysis
