# Author Correction: Identification of a differentiation-related prognostic nomogram based on single-cell RNA sequencing in clear cell renal cell carcinoma

**DOI:** 10.1038/s41598-023-34897-z

**Published:** 2023-05-15

**Authors:** Zhi-Nan Xia, Jing-Gen Wu, Wen-Hao Yao, Yu-Yang Meng, Wen-Gang Jian, Teng-Da Wang, Wei Xue, Yi-Peng Yu, Li-Cheng Cai, Xing-Yuan Wang, Peng Zhang, Zhi-Yuan Li, Hao Zhou, Zhi-Cheng Jiang, Jia-Yu Zhou, Cheng Zhang

**Affiliations:** 1 Department of Urology, The First Affiliated Hospital of Harbin Medical University, Harbin, 150001 China; 2 Department of Urology Andrology, Women’s Hospital School of Medicine Zhejiang University, Hangzhou, 310006 China; 3 Department of Urology, The Fourth Affiliated Hospital Zhejiang University School of Medicine, Yiwu City, 322000 China; 4 Xinjiang Second Medical College, Karamay City, 834000 China; 5 Zhejiang University School of Medicine, Hangzhou, 310058 China

Correction to: *Scientific Reports* https://doi.org/10.1038/s41598-022-15206-6, published online 29 June 2022

The original version of this Article omitted an affiliation for Cheng Zhang. The correct affiliations are listed below.

Department of Urology, The First Affiliated Hospital of Harbin Medical University, Harbin, 150001, China.

Zhejiang University School of Medicine, Hangzhou, 310058, China.

The original Article has been corrected.

